# Kunitz type protease inhibitor from the canine tapeworm as a potential therapeutic for melanoma

**DOI:** 10.1038/s41598-019-52609-4

**Published:** 2019-11-07

**Authors:** Shiwanthi L. Ranasinghe, Vanessa Rivera, Glen M. Boyle, Donald P. McManus

**Affiliations:** 1 Molecular Parasitology Laboratory, Immunology Department, QIMR Berghofer Medical Research Institute, Brisbane, Australia; 2 Cancer Drug Mechanisms Group, Cell & Molecular Biology Department, QIMR Berghofer Medical Research Institute, Brisbane, Australia

**Keywords:** Cancer immunotherapy, Cancer immunotherapy, Recombinant protein therapy, Recombinant protein therapy

## Abstract

Modulating the tumor microenvironment to promote an effective immune response is critical in managing any type of tumor. Melanoma is an aggressive skin cancer and the incidence rate is increasing worldwide. Potent protease inhibitors have recently been extensively researched as potential therapeutic agents against various cancers. EgKI-1 is a potent Kunitz type protease inhibitor identified from the canine tapeworm *Echinococcus granulosus* that has shown anti-cancer activities *in vivo*. In this study we show that EgKI-1 significantly reduced the growth of melanoma in the B16-F0 mouse model and was not toxic to normal surrounding tissue. Moreover, EgKI-1 treatment significantly reduced survivin expression levels and increased the CD8+ T cell population in draining axillary lymph nodes. Therefore, EgKI-1 potentially reduces tumor growth by inducing apoptosis and modulating the tumor microenvironment, and has potential for development as an intra-lesional treatment for melanoma.

## Introduction

Australia and New Zealand have the world’s highest incidence rates for melanoma. It is the third most common cancer diagnosed in Australians and accounts for 9–12% of all new recorded cancers diagnosed^1^. In total, 10% of people diagnosed with melanoma have life-threatening metastasis and tissue invasion even after the primary melanoma is surgically removed. The use of combined targeted therapies with surgery still remains inadequate and further research to investigate new treatments is vital.

EgKI-1, identified from the tapeworm *Echinococcus granulosus*, is a potent neutrophil elastase (NE) inhibitor^2^ that was shown to significantly inhibit breast cancer growth in the MDA-MB-231 mouse model^3^. NE secreted by neutrophils in the tumor microenvironment has been shown to promote tumor proliferation as well as reducing the activity of CD8+ T cells^4^. A preliminary study we undertook showed EgKI-1 inhibited human melanoma cell line (CJM) *in vitro*^3^; a result which led us to undertake further investigation using the B16-F0 murine tumour cell line commonly used as a research model in C57BL/6 mice to study human skin cancers^5^.

Lymph nodes, which contain lymphoid cells, are positioned throughout the body and play a crucial role in the immune system^6^. Tumor draining lymph nodes (TDLNs) induce anti-tumor T cell responses^6^; however there is a high susceptibility to melanoma induced suppression of CD8+ T cells^7^. Investigation of immune cells in the TDLNs can lead to a better understanding of the mechanisms involved in cancer progression and/or the development of improved cancer treatments.

In this study we determined the effect of EgKI-1 on lymphoid cells and innate cells in the spleen, axillary lymph nodes and tumor tissue using flow cytometry analysis. Local tumor treatment is particularly advantageous in the case of melanoma due to the low dose needed, the resulting decreased toxicity, and in minimising the risk of auto-immunity^6,8^. Furthermore, local targeting can overcome tumor-mediated immune cell suppression by activating robust anti-tumor T cell responses.

In addition, we investigated the modulation of different proteins at the mRNA level following EgKI-1 treatment. These included survivin, an apoptotic and mitotic regulator, that is overexpressed in melanoma and promotes melanoma metastasis^9^, matrix metalloproteins (MMPs), proteases that facilitate invasion, metastasis and regulate tumor cell proliferation and apoptosis^10^, and Bcl-2 (B-cell lymphoma 2), another regulator of apoptosis^11^.

## Results

**Figure 1:**
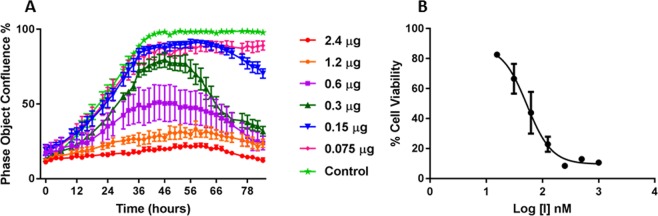
(**A**) Inhibition of the growth of B16-F0 cells *in vitro* with increasing concentrations of EgKI-1. (**B**) Inhibition curve of B16-F0 cells showing an IC_50_ of 53.24 nM. N = 3.

We assessed the potential of EgKI-1 as an anti-cancer agent for the treatment of melanoma using the B16-F0 mouse model. EgKI-1 inhibited the growth of B16-F0 cells in a dose-dependent manner *in vitro* (Fig. 1A) with an IC_50_ of 53.24 nM (Fig. 1B).

**Figure 2:**
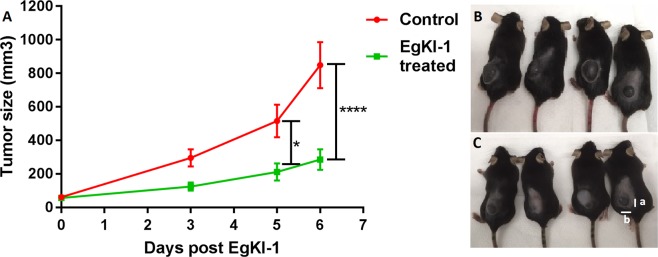
Intralesional EgKI-1 treatment prevents melanoma growth. (**A)** Melanoma growth in control and EgKI-1 treated mice over time. Intralesional EgKI-1 treatment (1.125 µM) was administered at 2, 4 and 6 days **(B)** Image showing the sizes of tumors in four of control and **(C)** treated mice at the end of the experiment (“a” indicates length and “b” indicates width). *For 0.005 ≤ p < 0.05, ****for p < 0.0001 according to 2-way ANOVA, with 95% confidence interval (CI). N = 6 in each group and experiments were repeated in duplicate.

In the *in vivo* mouse model of melanoma, intralesional treatment of established tumors with 1.125 µM EgKI-1 significantly slowed melanoma growth compared to the control group with a percentage tumor growth reduction of 68% (Fig. 2).

Fluorescence-activated cell sorting (FACS) analysis of various cell surface markers was carried out (Supplementary Fig. 1). The results showed that, 7 days after EgKI-1 treatment, the percentage of CD8+ killer (cytotoxic) T cells present in axillary LNs was significantly lower in the EgKI-1 treated mice compared with the control group.

**Figure 3:**
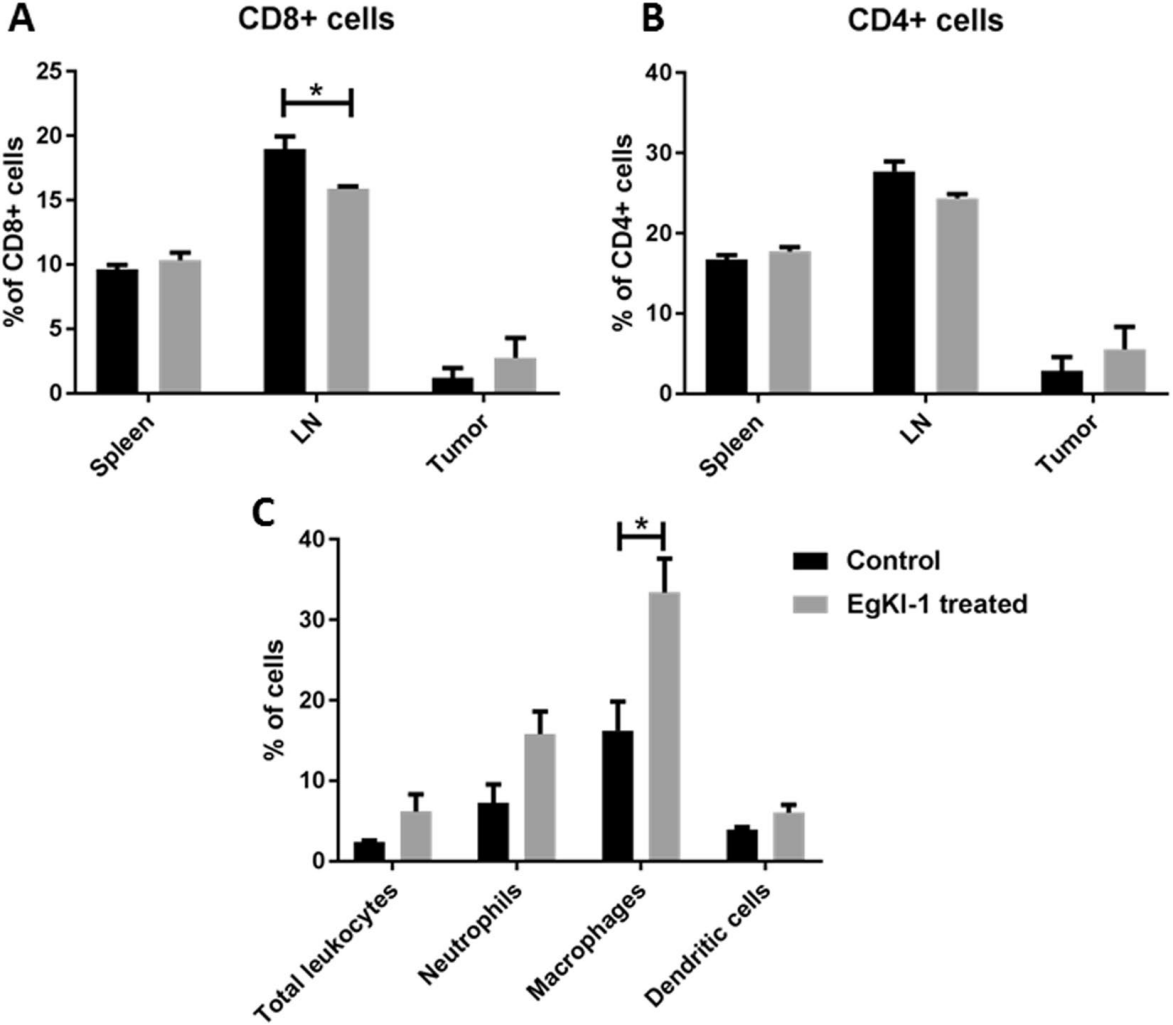
Percentage of T cells and innate cells in different tissues of control and EgKI-1 treated mice. (**A)** CD4+ and **(B)** CD8+ cells in spleen, lymph node and tumor tissue and **(C)** innate cells in tumor tissue after 7 days post- treatment with EgKI-1 (1.125 µM). *For 0.005 ≤ p < 0.05, 2-way ANOVA test with 95% CI. N = 6 in each group and experiments were repeated in duplicate.

This result favorably indicates improved drainage of CD8+ cells to the tumor tissue (Fig. 3A). However, there was no significant difference between the levels of CD4+ cells in control- and EgKI-1-treated mice (Fig. 3B). Considering innate immune cells there was a significant increase in the number of macrophages in the tumor tissue of EgKI-1 treated mice compared with the control mice (Fig. 3C). No significant differences were apparent in cytokine expression in the tumor tissue of treated and control mice determined using the Cytometric Bead Array (CBA) mouse Th1/Th2/Th17 cytokine kit (data not shown).

**Figure 4:**
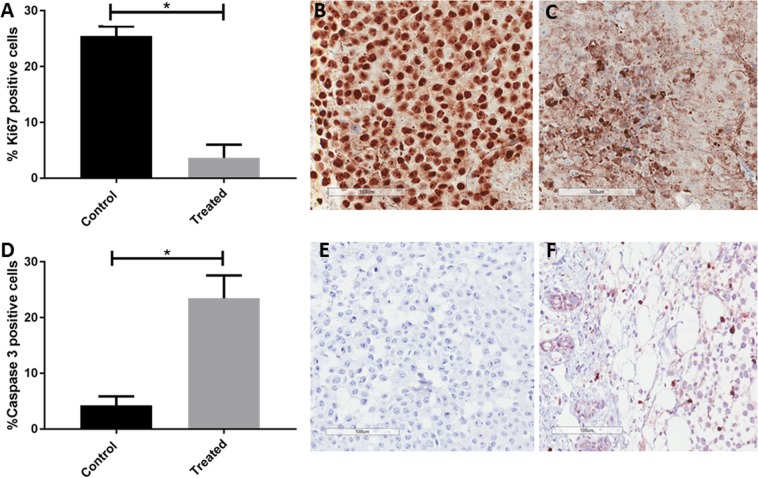
(**A)** Percentage of Ki67 positive cells in control and EgKI-1-treated tumor sections, microscopy images showing tumor tissue sections of **(B)** control and **(C)** treated mice **(D)** Percentage of caspase-3 positive cells in control and EgKI-1-treated tumor sections, microscopy images showing tumor tissue sections of **(E)** control and **(F)** treated mice, *for 0.005 ≤ p < 0.05 by student t-test with 95% CI. Scale bar indicates 100 µm. N = 3.

There was a significant reduction in Ki67 expression (Fig. 4A–C) in EgKI-1-treated tumor tissue compared with the controls indicating significantly less proliferation of melanoma cells in treated mice. Similarly, a significant increase of caspase-3 was evident in melanoma harvested from mice treated with EgKI-1 compared to controls (Fig. 4D–F). Hematoxylin & Eosin staining of EgKI-1-treated and control tumor sections indicated there was neither acute toxicity on tumor cells 24 hours after treatment nor toxicity after 7 days (Supplementary Fig. 2), indicating that EgKI-1 could be used as a therapeutic without adversely affecting normal surrounding cells.

**Figure 5:**
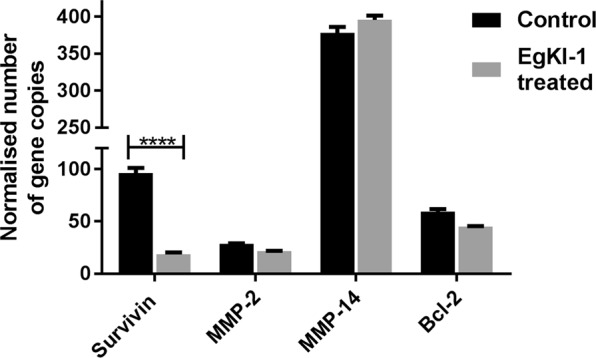
Normalised gene copy numbers for survivin, MMP-2, MMP-14 and Bcl-2 in EgKI-1(1.125 µM)-treated B16-F0 cells compared with control cells. ****For p < 0.0001 according to 2-way ANOVA, with 95% CI. N = 3.

qPCR analysis was carried out to investigate the role of EgKI-1 on different gene expressions mainly related to tumor growth. According to the results, EgKI-1 treatment significantly inhibited the expression of survivin in B16-F0 cells compared with the control non-treated cells (Fig. 5).

## Discussion

The results reported here indicate that EgKI-1 treatment was able to significantly decrease the growth *in vivo* of invasive B16-F0 melanoma in mice. Targeting the tumor microenvironment and TDLNs locally can significantly improve anti-tumor immunological processes^6^. Furthermore, local administration can significantly reduce possible toxicity and autoimmunity caused by systemic administration^6^.

Histological analysis of tumor sections indicated that no acute toxicity was generated by the local administration of EgKI-1. Survivin, which is an apoptotic and mitotic regulator, is usually overexpressed in melanoma. Research to date strongly supports a direct role for survivin in tumor metastasis^9^. Overexpression of survivin protects melanoma cells^12^ and survivin suppression is essential for EgKI-1 induced melanoma apoptosis. According to the ICH analysis EgKI-1 treated tumor sections express significantly higher levels of caspase-3, indicating melanoma cell apoptosis. Therefore, EgKI-1 not only directly induces tumor cell apoptosis but also indirectly via survivin suppression and thus shows promise as a potential new treatment against melanoma.

Ki67 staining indicated most of the melanoma cell proliferations were arrested, releasing molecules such as damage associated molecular patterns (DAMPs)/alarmins which promote and exacerbate the inflammatory response^13^. Tumor-resident CD8+ T cells are associated with improved survival of melanoma patients^14^. On the other hand, CD8+ cytotoxic T cells drain into the tumor from the axillary lymph node, as the TDLN for the melanoma. It has previously been shown that activated T cells in TDLNs of melanoma patients resulted in preferential apoptosis of human cancer cell lines^15^. A previous study showed that purified NE inhibits the transendothelial migration of T lymphocytes^16^. Accordingly, NE inhibition by EgKI-1^2^ may increase T cell migration to tumors, thereby facilitating an attack on tumor cells and favourably modulating the tumor microenvironment. However, more research is needed to confirm this feature *in vivo*. Macrophages in tissues arise from monocytes and have strong phagocytic potential^17^. Tumor-associated macrophages (TAMs) are key antigen presenting cells and have, apparently paradoxical properties, with the ability to both reduce and promote tumor growth^17^. However, if properly instructed, macrophages can mediate robust anti-tumor responses including eliminating malignant cells, inhibiting angiogenesis and depleting fibrosis^18^. EgKI-1 treatment possibly activates macrophages of the M1 phenotype to induce anti-tumor mechanisms^19^.

In conclusion, EgKI-1 has the potential to reshape immune regulation in tumors, induce apoptosis and inhibit cell proliferation leading to tumor growth inhibition. Thus, EgKI-1 is a promising molecule to develop as a topical treatment for melanoma. Additional studies should be undertaken with the EgKI-1 protein to further evaluate its potential to promote an inflammatory response as well as determining the mechanisms of direct cancer cell killing by this potent neutrophil elastase inhibitor.

## Methods

### B16-F0 cell growth analysis

Recombinant EgKI-1 protein was expressed and purified in yeast as described^3^. B16-F0 cells were cultured in complete media (RPMI-1640 supplemented with 10% (v/v) heat-inactivated fetal calf serum (Thermo Fisher Scientific, Waltham, USA), 3 mM 4-(2-hydroxyethyl)-1-piperazineethanesulfonic acid (HEPES)), 100 U/ml penicillin and 100 μg/ml streptomycin (Thermo Fisher Scientific). Cultured cells were routinely checked for mycoplasma infection by a specific PCR-based assay^20^ and were always negative.

The effects of EgKI-1 treatment on growth and migration of B16-F0 cells was assessed by Incucyte cell growth assay^3^. The experiment was repeated three times and the IC_50_ (the concentration of EgKI-1 needed to inhibit cell growth by 50%) value was calculated using “log (inhibitor) vs. response - Variable slope (four parameters)” under nonlinear regression analysis in GraphPad Prism 7 software.

### *In vivo* mouse model

B16-F0 cells were cultured and collected by trypsinization. After 3 washes with PBS, to remove any traces of medium, 8 × 10^5^ cells were suspended in 50 µl PBS. Then, 7–8 weeks old female C57BL/6 mice (7 each in the control and treatment groups) were injected with B16-F0 cells (8 × 10^5^ cells per mouse) subcutaneously into the dorsal flank region. Mice were carefully monitored and tumour size was measured by digital Vernier calliper every other day according to the formula, a × b × b × 0.5, where “a” the length and “b” the measured breadth of the tumor. When the tumour volume reach 50 mm^3^, intra-tumour EgKI-1 treatment (80 µg in 50 µl per mouse) commenced and was repeated every other day. The control group was treated with 50 µl buffer (150 mm NaCl, 20 mM Tris, pH 7). 24 hours after the initial treatment 3 mice from each group were sacrificed to check acute toxicity by analysing tumor histology as later described. Mice were also assessed using an approved clinical distress score sheet over the course of the experiment (Table 1). Scores for each parameter were summed to give a possible total of 8. Less than 3 was considered a mild clinical score, between 3–6 a moderate and over 6 was considered severe. Experimentation on an individual mouse was terminated when an unacceptable clinical score (>6) was reached, or the cumulative tumor burden of the animal exceeded 1000 mm^3^.

**Table 1:** Clinical scoring for Drug Treated and Tumor Bearing Mice.

Criteria	Grade 0	Grade 1	Grade 2
Weight loss	<10%	>10 to <25%	>25%
Posture	Normal	Hunching noted only at rest	Severe hunching impair movement
Activity	Normal	Mild to moderately decreased	Stationary unless stimulated
Fur texture	Normal	Mild to moderate ruffling	Severe ruffling/ poor grooming

Mice were humanely euthanized by exposure to carbon dioxide at the end of the experiment and samples were subjected to assess histology and immune responses. The experiment was repeated twice.

The mean reduction in tumour growth was calculated according to the formula^21^:

Mean reduction in tumour growth (%) = (mean tumour volume in control group – mean tumour volume in treated group) × 100/mean tumour volume in control group

Results were analysed using GraphPad Prism software with 2-way ANOVA.

### Histological analysis

Histological analysis of tumor sections from paraffin blocks, stained for hematoxylin and eosin (H&E), and the proliferation marker Ki67 were carried out as described previously^3^. Ki67 is a nuclear protein that is expressed in proliferating cells and is a recognized marker for cell proliferation in solid tumors^22^. Caspase-3 is an apoptosis marker of cells undergoing apoptosis^23^ and the immunohistochemistry protocol used in this study labels cells with activated caspase-3 presence. Briefly, histological melanoma sections (3–4 µm) of control and EgKI-1-treated mice were affixed to positively charged adhesive slides and air-dried overnight at 37 °C. Sections were then dewaxed and rehydrated through descending graded alcohols to Tris-buffered saline (TBS), pH 7.6, followed by incubation in 2.0% (v/v) H_2_O_2_ in PBS for 10 minutes to block the endogenous peroxidase activity. Slides were then subjected to an oxidizing step with 0.3% (w/v) aqueous potassium permanganate and 3.0% (w/v) aqueous oxalic acid to bleach melanin, which can obscure the caspase-3 signal. A heat antigen retrieval step was then carried out with Tris/EDTA buffer pH 8.8 for 15 minutes at 105 °C using a Biocare Medical Decloaking Chamber. Normal goat serum (10%) was then applied for 30 min and rabbit anti-cleaved Caspase-3 antibody (1:100) (Biocare Medical) applied for 2 hours at room temperature. Following 3 washes with TBS, an anti-rabbit MACH1 Universal secondary antibody (Biocare Medical) was applied for 30–45 minutes. After another 3 washes with TBS, color was developed with 3,3′-diaminobenzidine (DAB) for 5 to 10 minutes. Sections were then washed using gently running tap water for 5–10 minutes to remove excess chromogen and lightly counterstained in haematoxylin, then dehydrated through ascending graded alcohols, cleared in xylene, and mounted using DePeX.

Staining of the slides was carried out in the Histology Facility at QIMR Berghofer Medical Research Institute. Stained slides were scanned at 40× magnification with an Aperio Scanscope XT slidescanner and digital images were analyzed with ImageScope viewing software. The Aperio nuclear algorithm, which is based on the spectral differentiation between brown (positive) and blue counter staining, was used for analysis of Ki67 and caspase-3 signals. Total percentage positivity for each slide was then calculated and statistically analysed using GraphPad Prism software.

### Analysis of immune reactions

Single cell suspensions were made from spleen and axillary lymph nodes. Isolated tumor tissue was filtered through 70 µm filter and subjected to magnetic isolation of immune cells using CD45 MACS microbeads according to manufacturer’s instructions (Militeniy Biotech, Macquarie Park, NSW, Australia). Single cells were then stained to analyse lymphocytes (CD8, CD4 and B cells) and innate cells (neutrophils, macrophages, natural killer cells and dendritic cells) with FACS using two flow cytometry cocktail panels and a Fortessa B flow cytometer (BD Biosciences, Franklin Lakes, New Jersey).

Cocktail I was used to identify lymphoid cell populations and included: CD3-BV421, CD4-AF700, CD8- BV650, CD19-FITC and viability stain FVS780.

Cocktail II was used to identify innate immune cell populations and included: CD11b-PerCPcy5.5, Ly6C-FITC, Ly6G-PE, MHC II-BV711, F4/80-BV421, CD11c-APC and viability stain FVS780.

Cells were then incubated for 30 min on ice in the dark followed by a washing step before they were fixed in cytofix buffer (BD Biosciences). After 20 min incubation on ice, cells were washed with FACS buffer (200 µl of PBS + 2% fetal calf serum (FCS)) and resuspended in 200 µl of FACS buffer. Stained samples were analysed using a 4-laser LSR Fortessa (BD Biosciences) and analysed with FACS Diva and FlowJo software. For the analysis, debris was excluded based on size (forward scatter, FSC) and complexity (side scatter, SSC). Doublets and triplets were further excluded based on FSC height and FSC area. Viable cells which were negative for the FVS780 were then analysed to identify different cell populations as described^24^.

Serum was separated from mouse blood samples collected by heart puncture at the termination of each mouse model experiment. Samples were then used to determine cytokine levels using a Cytometric Bead Array (CBA) mouse Th1/Th2/Th17 cytokine kit (BD Biosciences) which measures IL-2, IL-4, IL-6, IFNγ, TNFα, IL-17A and IL-10 levels in a single sample. The sensitivity of the kit ranges from 5 to 20 pg/ml depending on the cytokine measured.

### Quantitative PCR analysis

Quantitative real-time PCR (qPCR) analysis was carried out to investigate the role of EgKI-1 treatment on gene expression in melanoma cells. B16-F0 cells were grown to 70% confluency in 6 well plates and treated with EgKI-1 at a concentration of 1.125 µM. 48 hours post-treatment approximately 1 × 10^6^ cells were collected by trypsinisation, washed 3× with PBS and stored at −20 °C until use. RNA was extracted from the cells using a RNeasy mini kit (QIAGEN, Hilden, Germany) according to the manufacturer’s instructions. Eluted RNA was then analysed by nanodrop and used to synthesise cDNA using a QuantiTect Reverse Transcription kit (QIAGEN). The cDNA samples were then stored at −20° for qPCR analysis.

The expression levels of the genes associated with melanoma: Matrix metalloproteinase 2 and 14 (MMP-2 and MMP-14), surviving and Bcl-2, were analysed by qPCR using the Rotor-Gene Q series software. GAPDH (glyceraldehyde 3-phosphate dehydrogenase) was used as the internal standard^25^. The sequences of the primers used are given in Table 2. Utilizing control non-treated melanoma cells as calibrator, the relative quantitative values of target genes were expressed as 2^−ΔΔCt^ values. Briefly, qPCR analysis was performed with 150 ng of total cDNA and QuantiNova SYBR green PCR master mix (QIAGEN) in a 20 µl reaction volume. All cDNA samples were subjected to qPCR three times using a Corbett Rotor Gene 6000 thermal cycler. Results were statistically analysed with GraphPad Prism software.

**Table 2:** Sequences of forward and reverse primers for the genes analysed by qPCR.

Gene	Forward primer	Reverse primer
MMP-2	GACGGTAAGGACGGACTC	ACTTCACACGGACCACTT
MMP-14	CGAGGTGCCCTATGCCTAC	CTCGGCAGAGTCAAAGTGG
Bcl-2	AGTACCTGAACCGGCACCT	CTTCAGAGACAGCCAGGAGAA
Survivin	CAGATTTGAATCGCGGGACCC	CCAAGTCTGGCTCGTTCTCAG
GAPDH	ACCCACTCCTCCACCTTTGAC	TGTTGCTGTAGCCAAATTCGTT

### Animal ethics statement

This study was performed in strict accordance with protocols approved by the Animal Ethics Committee of QIMR Berghofer Medical Research Institute (Approval Number A1606-617M) which adheres strictly to the Australian code of practice for the care and use of animals for scientific purposes, as well as the Queensland Animal Care and Protection Act 2001, and Queensland Animal Care and Protection Regulation 2012. Mice were housed in a specific pathogen-free animal facility at the QIMR Berghofer with 12 hours light/dark cycle and continual access to water and food.

### Statistical analysis

All data are presented as the means ± standard mean of error (SEM) of three individual experiments unless otherwise stated. A p-value of <0.05 was considered as statistically significant according to the 2-way ANOVA and Student’s t-tests. GraphPad Prism version 7 was used for all statistical analysis.

## Supplementary information

Supplementary material
