# Supplementary material for: Kunitz type protease inhibitor from the canine tapeworm as a potential therapeutic for melanoma
Source: Sci Rep. 2019 Nov 7;9:16207. doi: 10.1038/s41598-019-52609-4 (PMC6838156; doi:10.1038/s41598-019-52609-4)
Supplement: Supplementary file 1 — Supplementary material [file 41598_2019_52609_MOESM1_ESM.pdf]

Kunitz type protease inhibitor from the canine tapeworm as a potential therapeutic for melanoma

Shiwanthi L Ranasinghe[*1], Vanessa Rivera[1], Glen M Boyle[2], Donald P McManus[1]

[1] Molecular Parasitology Laboratory, Immunology Department, QIMR Berghofer Medical Research Institute, Brisbane, Australia

[2] Cancer Drug Mechanisms Group, Cell & Molecular Biology Department, QIMR Berghofer Medical Research Institute, Brisbane, Australia

*shiwanthi.ranasinghe@qimrberghofer.edu.au

Supplementary Figure 1:

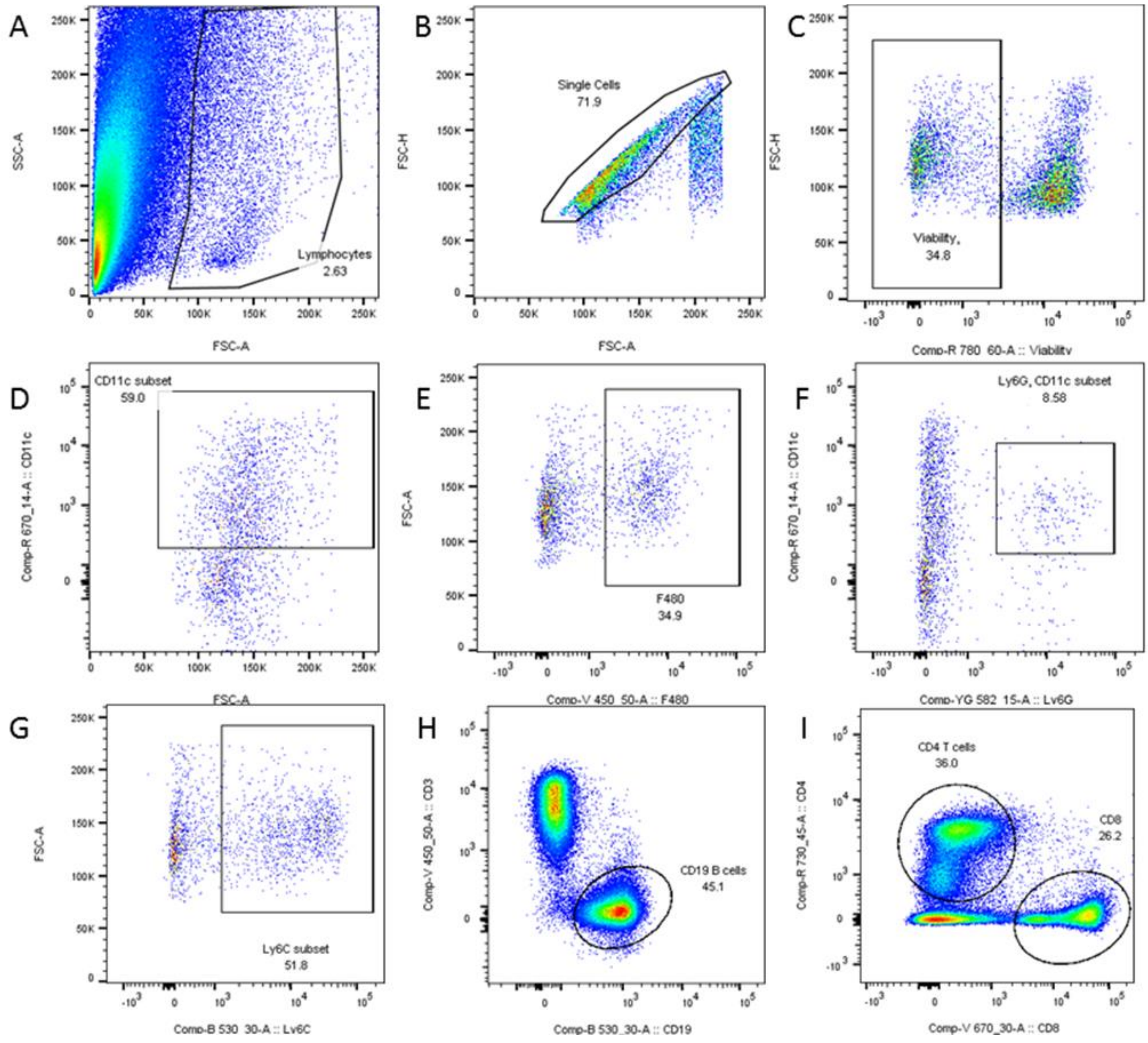

Supplementary Figure 1: FACS analysis with surface cell markers. Isolation of **(A)** lymphocytes **(B)** single cells **(C)** viable cells **(D)** CD11c subset **(E)** F4/80+ macrophages, **(F)** Ly6G+ neutrophils and **(G)** Ly6C+ eosinophils from CD11b+ subpopulation of viable single cells with cocktail 2 staining. Isolation of **(H)** CD19 positive B cells, **(I)** CD8+ and CD4+ cells from CD3 subset using flow cytometry staining cocktail 1.

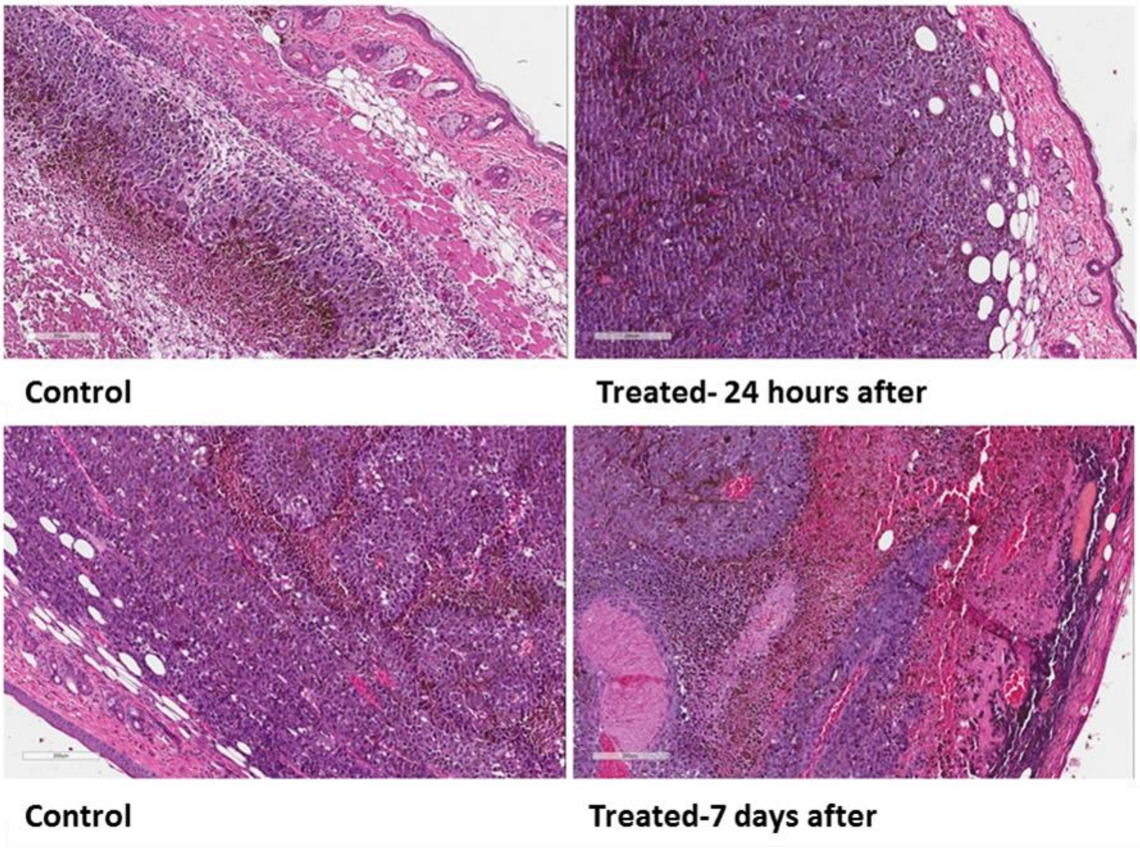

Supplementary Figure 2: Hematoxylin and Eosin stained tumour tissue sections (×10) of control and treated mice 24 hours (above) and 7 days after (below) treatment. Scale bar indicates 100 µm.
